# Monte Carlo samplers for efficient network inference

**DOI:** 10.1371/journal.pcbi.1011256

**Published:** 2023-07-18

**Authors:** Zeliha Kilic, Max Schweiger, Camille Moyer, Steve Pressé

**Affiliations:** 1 Department of Structural Biology, St. Jude Children’s Research Hospital, Memphis, Tennessee, United States of America; 2 Center for Biological Physics, ASU, Tempe, Arizona, United States of America; 3 Department of Physics ASU, Tempe, Arizona, United States of America; 4 School of Mathematics and Statistical Sciences, ASU, Tempe, Arizona, United States of America; 5 School of Molecular Sciences, ASU, Tempe, Arizona, United States of America; University of Pittsburgh, UNITED STATES

## Abstract

Accessing information on an underlying network driving a biological process often involves interrupting the process and collecting snapshot data. When snapshot data are stochastic, the data’s structure necessitates a probabilistic description to infer underlying reaction networks. As an example, we may imagine wanting to learn gene state networks from the type of data collected in single molecule RNA fluorescence *in situ* hybridization (RNA-FISH). In the networks we consider, nodes represent network states, and edges represent biochemical reaction rates linking states. Simultaneously estimating the number of nodes and constituent parameters from snapshot data remains a challenging task in part on account of data uncertainty and timescale separations between kinetic parameters mediating the network. While parametric Bayesian methods learn parameters given a network structure (with known node numbers) with rigorously propagated measurement uncertainty, learning the number of nodes and parameters with potentially large timescale separations remain open questions. Here, we propose a Bayesian nonparametric framework and describe a hybrid Bayesian Markov Chain Monte Carlo (MCMC) sampler directly addressing these challenges. In particular, in our hybrid method, Hamiltonian Monte Carlo (HMC) leverages local posterior geometries in inference to explore the parameter space; Adaptive Metropolis Hastings (AMH) learns correlations between plausible parameter sets to efficiently propose probable models; and Parallel Tempering takes into account multiple models simultaneously with tempered information content to augment sampling efficiency. We apply our method to synthetic data mimicking single molecule RNA-FISH, a popular snapshot method in probing transcriptional networks to illustrate the identified challenges inherent to learning dynamical models from these snapshots and how our method addresses them.

## Author summary

Decoding biochemical reaction networks–the number of species, reactions connecting them and reaction rates–from snapshot data, such as data drawn from single molecule fluorescence *in situ* hybridization (smFISH) experiments, is of critical interest. Yet, this task’s challenges are currently addressed with network specification heuristics since: 1) network size cannot be specified independently of rates; 2) rates may be separated by orders of magnitude, generating stiff ODEs; 3) uncertainty is not propagated into networks and parameters. We present, inspired by recent computational statistics tools, a method to simultaneously deduce reaction networks and associated rates from snapshot data while propagating error over all unknowns. We achieve this by treating network models as random variables and, within the Bayesian nonparametric paradigm, develop a posterior over models themselves. This multidimensional posterior naturally contains multiple hills and valleys, so we propose a combination of samplers allowing for the first simultaneous and self-consistent inference of networks and their associated rates. Our method’s ability to treat arbitrary numbers of states contrasts the current state of the art and may modify previous biological conclusions based on network-determination heuristics. We demonstrate on method to synthetic data mimicking smFISH experiments and demonstrate its improvement over naive MCMC schemes.

## 1 Introduction

Systems and Computational Biology model allosteric enzyme control [1], chromatin re-organization [2], transcriptional regulation [3], metabolic interactions [4] and tumor growth [5], among other phenomena, with reaction networks. Ideally we could learn the structure of reaction networks by observing them continuously in time. However, this is often either impossible or introduces additional complications to the required model.

For example, actively monitoring transcription by fluorescence may introduce delays arising from maturation of co-translationally generated protein and label [6]. Also, since single molecule monitoring of transient gene expression dynamics in continuous time requires tracking diffraction-limited actively diffusing RNA, these methods are limited to low-expression genes to avoid crowded tracking [7]. Meanwhile, deducing a reaction network for the expressed genes may only require levels of RNA at various time points [8].

In addition to errors incurred in monitoring dynamics in real-time, in some cases, it may be altogether unnecessary to have time-resolved information [9], for example when probing steady-state reaction network dynamics.

Indeed, sufficient statistics might be accessed by interrupting an ensemble of system instances (*e.g.*, fixing individual cells) and recording instantaneous data from them at successive times. We refer to data collected in this manner as ‘snapshot data’. For example, if we wish to infer gene networks, we may randomly select a subpopulation of cells at various time points and subsequently lyse or fix them to enumerate their RNAs [10].

Promising methods using snapshot acquisition exist across the biological sciences, including: pulse-chase [11, 12], sequencing experiments (RNAseq) [13–17], single molecule Fluorescence *In Situ* Hybridization (smFISH) [18, 19] and associated methods of RNA quantification such as expansion microscopy [20]. As such, developing analytical methods to deduce reaction networks suiting snapshot data is of primary concern to Systems and Computational Biology.

While in practice the reaction network, its connectivity between nodes, and its associated rates are encoded in the data, extracting this information has presented unique challenges [21]. Time scales separating rate values may be considerable [9] and reaction networks with rates spanning a broad set of time scales increases the stiffness of the resulting differential equations [22, 23]. Furthermore, multiple network structures and reaction rate combinations may fit the data almost equally well (put otherwise, different networks describing observations similarly well may have drastically different rates) [24]; additionally, even for a known network, multiple parameter combinations may fit the data equally well (parameters are highly correlated due to additive changes introduced in gene expression) [9]. In fact, owing to these complications, multiple models may be comparably probable *a posteriori*, a characteristic we later refer to as ‘approximate model indeterminacy.’

Within a Bayesian inference setting to estimate reaction networks, these complications introduce hills and valleys in the multidimensional posterior over models naturally leading to the computational demise of naive Markov-Chain Monte Carlo (MCMC) methods [25] that have so far been limited to decoding rates for hand-specified nodes and connectivities between nodes [3, 24, 26, 27]. Within the current MCMC methods, determining the underlying reaction network or calibrating certain rates or time-dependencies may require additional control experiments leading to prohibitive experimental overhead [3] or a prohibitive number of core-hours on computing clusters [28]. Working within a Bayesian nonparametric setting where the number of nodes (not just the reaction rates connecting a known number of nodes) are to be inferred, we exchange these problems with more tractable ones, addressable by modern computational inference techniques. As we will see shortly, we use nonparametrics by employing Beta-Bernoulli process priors [29–33] to learn networks, using a selection of advanced sampling techniques identified below.

To overcome difficulties presented by rates spanning orders of magnitude and unknown number of nodes and their connectivities, we systematically explore combinations of MCMC samplers. That is, in order to resolve the stiffness of ODEs involved in likelihood calculation, we employ Adaptive Metropolis-Hastings (AMH) to propose new samples based on previous ones; in order to propose probable parameters based on the local geometry of the posterior, we employ Hamiltonian Monte Carlo (HMC), avoiding the overhead incurred by costly computational or experimental rate or model calibration; in order to maximize the explored region of the space of models, we package the above sampling schemes within a Parallel Tempering (PT) MCMC algorithm, allowing our method to characterize the posterior in a reasonable number of samples. Each of these methods and their particular advantages within the Bayesian paradigm are outlined in greater detail in Materials and Methods.

Our combination of sampling techniques provides increased efficiency which turns out to be critical in estimating network node numbers and rates simultaneously and self-consistently, going beyond existing state-of-the-art rate inference techniques [3, 25, 34]. To demonstrate this, we tested our sampling algorithm on a range of biologically realistic networks and rate values and assessed the added efficiency of each novel sampling technique, by replicating our analysis with each method omitted. We demonstrate that only our full method can simultaneously learn reaction rates and network structures at high sampling efficiency for a variety of underlying networks [35].

## 2 Materials and methods

### 2.1 A concrete example of a biochemical network

**Fig 1:**
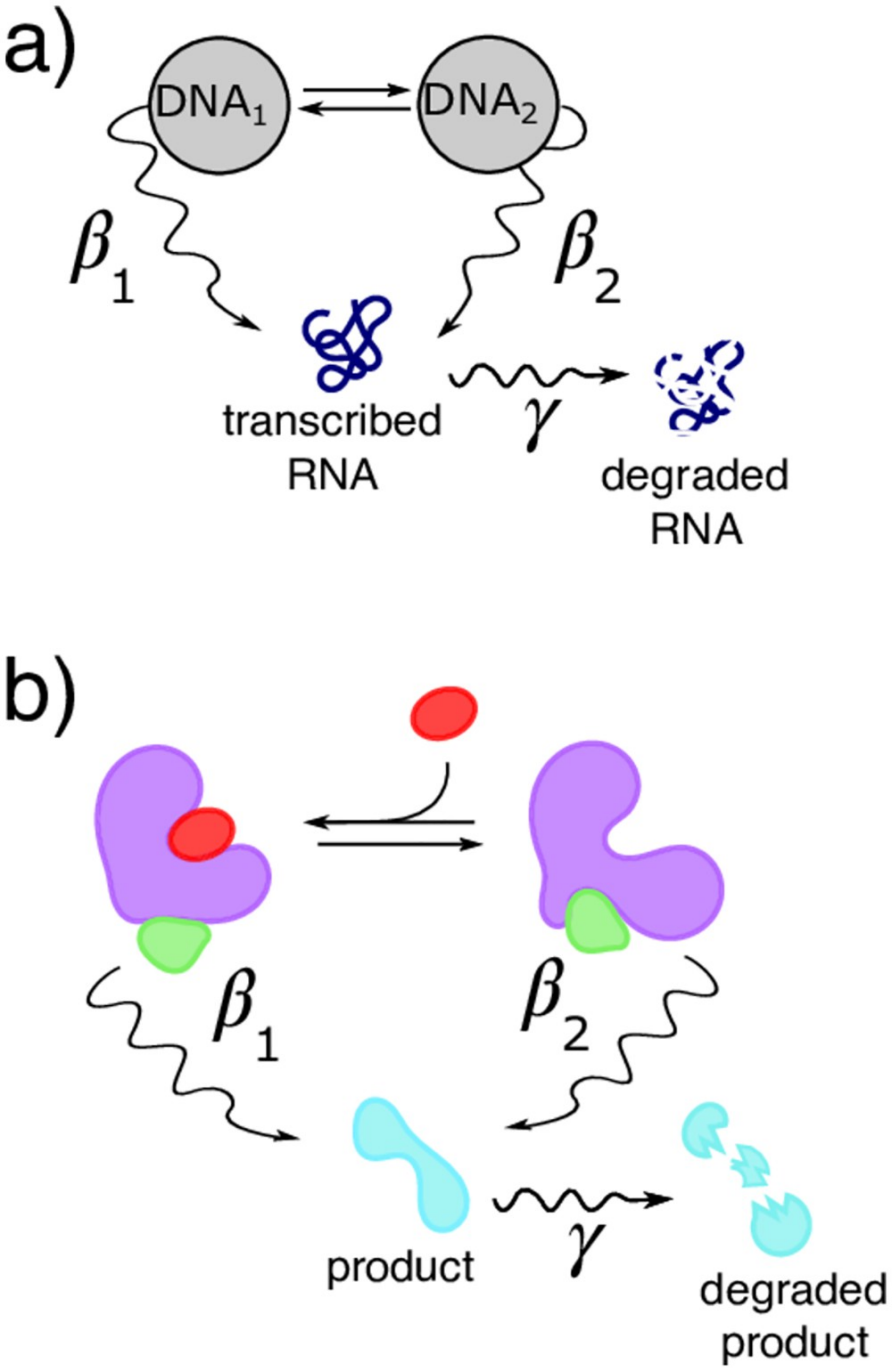
Two examples of reaction networks. In both networks, straight arrows illustrate possible transitions between network states. Other arrows depict additional biochemical reactions such as production rates (with rate *β*) or degradation rates (with rate *γ*). For example, **a)** depicts a two state gene network. Grey circles depict RNA production states, differentiated by their different production rates [36]. Alternatively, **b)** depicts enzyme catalysis under allosteric enzyme control [37]. Production states represent the enzyme with or without a bound repressor. Both are outlined in greater detail in section 1.1 in S1 Text.

Here we focus on deducing reaction network models, termed ‘gene networks’, from snapshot RNA count data inspired by smFISH experiments. A gene network is fully specified upon determining the number of nodes (gene states), and the strength of their connections (RNA transcriptional rates in each state, the transition rates between gene states distinguished by differing RNA production rate, and a global RNA degradation rate); see Fig 1A.

We will elaborate on the precise interpretation of each parameter in Fig 1’s caption, as well as describe rate interpretation for an alternative system modeled by a precisely the same biochemical reaction network described here in section 1.1 in S1 Text.

### 2.2 Data generation

An *N* state gene network is completely specified by its gene states *σ*_1_,…,*σ*_N_, their various RNA transcription rates $\bar{\beta }=(\beta_{1},\beta_{2},\ldots ,\beta_{N})$, rates of gene state transitions $\bar{k}=\left(k_{\sigma_{l}\to \sigma_{l}}\right)$ for *l* = 1, …, *N*, *l*′ = 1, …, *N* with *l* ≠ *l*′ and the overall RNA degradation rate *γ*. We refer to all dynamical rates compactly as $\theta =(\bar{k},\bar{\beta },\gamma )$. With these parameters at hand, we simulate Gillespie trajectories with duration *t*_1:*K*_ and record their final state $m_{t_{1:K}}^{j}$. To generate a full set of observations we then repeat this procedure *J*_*k*_ times for each *t*_*k*_. In this manner, we simulate snapshot data mimicking smFISH experiments [3, 24–26, 38–40] by collecting simulated RNA count data from *J*_*k*_ cells at all time points *t*_*k*_, and a full set of data is $D=\bar{\bar{m}}={\left\{{\left\{m_{t_{k}}^{j}\right\}}_{j=1:J_{k}}\right\}}_{k=1:K}$.

### 2.3 The inference problem

Now given data $D=\bar{\bar{m}}$, our goal is to estimate the model (in the smFISH case, determined by the number of gene states *N*) and its parameters *θ*. We accomplish this within a Bayesian nonparametric framework by drawing samples from the posterior, $P(N,\theta |\bar{\bar{m}})$. By Bayes’ theorem this posterior is given by

$$\begin{array}{c} P\left(N,\theta |\bar{\bar{m}}\right)\propto P\left(\bar{\bar{m}}|N,\theta \right)P\left(N,\theta \right). \end{array}$$ (1)

We therefore begin constructing the posterior by outlining calculation of the likelihood $P(\bar{\bar{m}}|N,\theta )$, followed by outlining our chosen priors *P*(*N*, *θ*) and finally describing our sampling method.

#### 2.3.1 Likelihood

The Chemical Master Equation (CME)

$$\begin{array}{ccc} \frac{d}{dt}P(t|\theta ) & = & AP(t|\theta ) \end{array}$$ (2)

is specified for a given model by the structure of the generator matrix **A** (in order maintain our focus on the likelihood’s structure, we give the exact form of **A** later in section 1.2 in S1 Text) and an initial probability vector over system states **P**(*t* = 0|*θ*). The generator matrix dictates the reaction network and informs the time-varying probability vector **P**(*t*|*θ*), whose elements are probabilities over each possible state of the network.

Due to the nature of the transcription induction experiments whose output we wish to simulate [3,25,26], we assume that all cells are initially in the same gene state σ* with zero RNA and subsequently learn σ*. However, in general, *P*(*t* = 0|*θ*) can be specified as any probability vector, depending upon the initial experimental conditions at hand.

As we are more concerned here with sampling methods than the specific dynamics of any one reaction network, we restrict ourselves to a general discussion of the likelihood, though we enumerate **A**’s elements for each model of interest in section 1.2 in S1 Text.

Given the CME’s solution, the likelihood contribution for a set ${\bar{m}}_{k}$ of snapshot data arising at time *t*_*k*_ is formed from the independent product from cell *j* = 1 to cell *J*_*k*_ over cells and then product over times *t*_*k*_ from *k* = 1 to *K* as follows:

$$\begin{array}{ccc} P(\bar{\bar{m}}|N,\theta ) & = & \prod_{k=1}^{K}\prod_{j=1}^{J_{k}}(\sum_{s_{k}^{j}=\sigma_{1},\cdots ,\sigma_{N}}P_{\theta }^{t_{k}}(s_{k}^{j},m_{k}^{j})). \end{array}$$ (3)

#### 2.3.2 Priors on network nodes

With the likelihood at hand, we can construct our posterior probability distribution, $P(\theta |\bar{\bar{m}})$, by first specifying an independent prior on each parameter. Due to the possible scale separation in the rates, we consider identical independent log-normal priors on each element of *θ*. Since, in general, rates are quite distinct from one another, identical independent priors on each rate help ensure that recovered ground-truth rates in simulated data analysis do not result from tuning the prior. We select identical numerical values for the hyper-parameters for all priors (they are shown in table A in S1 Text). The reason the priors themselves have limited impact on our rate posteriors is for the simple reason that data is abundant, *i.e.*, the log-posterior contains one log-prior term and ∑_*k*_*J*_*k*_ additive log terms in the likelihood.

Alongside the rates, we can learn the number of states by introducing a number (*L*) of hypothetical states *σ*_1_,…,*σ*_*L*_ to the model, and assigning two parameters to each state. The first is a Bernoulli distributed (binary) parameter *b*_*l*_ (called a ‘load’) which indicates whether a given state is necessary. When *b*_*l*_ = 1, *σ*_*l*_ contributes to the generator matrix as described in section 1.2 in S1 Text. When *b*_*l*_ = 0, we skip over *σ*_*l*_ when constructing the generator matrix, eliminating its contribution to the likelihood. Second, a real hyper-parameter *q*_*l*_ ∈ (0, 1) (called a ‘success probability’) gives the *a priori* probability of sampling *b*_*l*_ = 1. Taken together, this scheme is called a Beta-Bernoulli process prior [29, 30], and its equations are as follows:

$$\begin{array}{ccc} q_{\ell } & ∼ & \text{Beta}(\frac{\zeta }{L},\frac{L-1}{L})\\ b_{\ell }|q_{\ell } & ∼ & \text{Bernoulli}(q_{\ell }) \end{array}$$

for *l* = 1, 2, …, *L* with $\bar{b}=(b_{1},b_{2},...,b_{L})$ and $\bar{q}=(q_{1},q_{2},...,q_{L})$.

Notably, describing each model by its number of states *N* as above simply requires assigning $N=\sum_{\ell =1}^{L}b_{\ell }$.

In this manner, by iteratively sampling $\bar{b},\bar{q}$ and *θ* using a Gibbs sampling scheme [41], we sample the appropriate number of states attributed to the gene network under observation at each iteration, rigorously overcoming the overfitting problem [42, 43]. Vitally, from this Gibbs scheme, we sample the number of states and all parameters from the **fully joint posterior over rates and models simultaneously, conditioned on the data**.

#### 2.3.3 Sampling from conditional posterior distributions

The Gibbs sampling scheme to sample the posterior on each parameter (*i.e.*, all elements $\theta ,\bar{b},\bar{q}$) divides each MCMC iteration into steps wherein a parameter or set of parameters are drawn from their respective conditional posteriors. Once each parameter ($\theta ,\bar{b},\bar{q}$) has been sampled from its conditional posterior, a single MCMC iteration is complete [42, 43]. We now outline the conditional posteriors required in each step of the Gibbs scheme as follows:

1. **Loads** ($\bar{b})$ Each load is sampled directly from its Bernoulli posterior, conditioned upon the most recent estimates of the remaining parameters (including the remaining loads, $\bar{b}\backslash b_{\ell }$ when sampling *b*_*ℓ*_) and on the data D
  $$\begin{array}{c} P\left(b_{\ell }|\bar{b}\backslash b_{\ell },\bar{q},\sigma^{*},\theta ,D\right)\propto P\left(D|\bar{q},\theta ,\bar{b}\right)P\left(b_{\ell }|\bar{b}\backslash b_{\ell },\bar{q},\sigma^{*}\right) \end{array}$$ (4)
  where the normalization constant of the above follows from $P\left(b_{\ell }=0|\bar{b}\backslash b_{\ell },\bar{q},\sigma^{*},\theta ,D\right)+P\left(b_{\ell }=1|\bar{b}\backslash b_{\ell },\bar{q},\sigma^{*},\theta ,D\right)=1$.
2. **Success Probabilities** ($\bar{q}$) Success probabilities are sampled using Metropolis-Hastings with Beta distributed proposals [31, 44–47].
3. **Initial Condition Gene State** (σ*) The initial condition gene state is sampled directly from its categorical posterior.
4. **Rates** (*θ*) Rate quantification is the most difficult Gibbs step (meaning that rates take the largest number of samples to reach convergence to ground truth). As such, rate quantification conducted using Metropolis sampling is expanded upon below.

#### 2.3.4 Parallel tempering

PT is a scheme wherein multiple MCMC chains draw samples from posterior probability distributions,

$$\begin{array}{c} P{\left(\theta |D\right)}_{h}={\left(P(D|\theta )P(\theta )\right)}^{\omega_{h}} \end{array}$$ (5)

where the temperature parameter for the *h*th chain, *ω*_*h*_ = 1/*T*_*h*_, is dictated by its temperature, *T*_*h*_, for *h* = 1, 2, …, *H* [48, 49]. Temperatures monotonically increase for each chain such that *T*_1_ = 1 < *T*_2_ < ⋯ < *T*_*h*−1_ < *T*_*h*_. In this way, the posterior probability distribution associated to the first chain is the target of inference, while the higher temperature chains merely enable movement of this principle chain around the space of models. Chains sampled from posterior probability distributions at higher temperatures explore the model space more easily due to the effective ‘flattening’ arising from higher temperatures.

We initialize each chain at random from its prior distribution. Then, each chain conducts a preset number of sampling iterations from the respective tempered posterior probability distributions [50, 51] as shown in 5. Then the final parameter set for each chain, following each round of sampling iterations, is probabilistically ‘swapped’ with another temperature using an MH variant with deterministic proposals. Concretely, if we propose a swap after a fixed number of iterations between the parameter sets from chain *a*, *θ*_*a*_, and from chain *b*, *θ*_*b*_, with *ω*_*a*_ > *ω*_*b*_, the proposal distribution is

$$\begin{array}{ccc} Q_{\theta_{a}^{old},\theta_{b}^{old}}\;(\theta_{a}^{prop},\theta_{b}^{prop}) & = & \delta_{\theta_{a}^{old}}\;(\theta_{b}^{prop})\;\delta_{\theta_{b}^{old}}\;(\theta_{a}^{prop}), \end{array}$$ (6)

where $\delta_{\theta_{i}}(\theta_{j})$ is the Kronecker delta function equal to 1 if all corresponding elements of *θ*_*i*_ and *θ*_*j*_ match and 0 otherwise, and the probability of accepting the swap is

$$\begin{array}{ccc} R_{\theta_{a}^{old},\theta_{b}^{old}}(\theta_{a}^{prop},\theta_{b}^{prop}) & = & \text{min}(1,e^{\left(\omega_{a}-\omega_{b})(P(\theta_{a}|D)-P(\theta_{b}|D)\right)}). \end{array}$$ (7)

The pairing of chains is done at random for each round maximizing the mixing amongst chains. Additionally, we optimize the swapping of chains by employing an adaptive parallel tempering algorithm for which we tune the temperature of each chain, except for the lowest temperature, according to the scheme from [52].

#### 2.3.5 Rate inference

Now that we have discussed the core Gibbs sampling scheme and the overall PT, we discuss how to evolve rates for chains at each temperature.

To obtain Metropolis-Hastings proposals for rates, our method supplements a traditional Adaptive Metropolis-Hastings (AMH) sampling scheme with the intermittent use of Hamiltonian Monte Carlo (HMC) sampling [53, 54]. Our AMH algorithm follows the adaptive scheme described in [55]. Briefly, each parameter set *θ* is updated subject to a Normal proposal distribution with an adaptable covariance matrix, **Σ**:

$$\begin{array}{c} Q_{\theta^{old}}(\theta^{prop})=\text{Normal}(\theta_{i}^{prop};\theta_{i}^{old},\Sigma ) \end{array}$$ (8)

where $\theta_{i}^{old}$ is the set of parameters associated to the previous iteration, and *θ*^*prop*^ is the proposed set of parameters. Once a proposal has been made, the new set is either accepted with probability

$$\begin{array}{c} R_{\theta^{old}}(\theta^{prop})=\text{min}(1,\frac{P(D|\theta^{prop})\;P\;(\theta^{prop})Q_{\theta^{prop}}\;(\theta^{old})}{P(D|\theta^{old})\;P\;(\theta^{old})Q_{\theta^{old}}\;(\theta^{prop})}), \end{array}$$ (9)

or rejected with the complementary probability ($1-R_{\theta^{old}}(\theta^{prop})$), and the algorithm repeats in an iterative fashion. The adaptation portion of the algorithm uses the movement of each parameter up to the current iteration to tune the covariance matrix, **Σ**. Concretely, an element of the covariance matrix, **Σ**_*ij*_, is specified as the covariance between parameters *i* and parameter *j* resulting from the previous sample sequences of both parameters. Then, in order to guarantee that the resulting matrix is a valid parameter for the multivariate normal proposal distribution, we add a small fixed multiple (*ϵ* = 3.7508 × 10^−17^) of the identity, to guarantee that the proposal covariance can be inverted when sampling [55, 56].

While this adaptive scheme incorporates correlations between parameters into the proposal covariance matrix, it fails to explicitly leverage the shape of the posterior probability distribution to inform proposals. Therefore, the HMC proposal method is introduced [57], to which AMH can be seen as a less effective, albeit less expensive analogue.

HMC generates proposals by reversibly evolving parameters according to pseudo-Hamiltonian dynamics [57]. We start with our target distribution

$$\begin{array}{c} P\;(\theta |\text{data})=\int dpP\;(\theta ,p|\text{data}) \end{array}$$ (10)

where the auxiliary variables *p* are introduced allowing us to write

$$\begin{array}{c} P\;(\theta ,p|\text{data})=\text{exp}(-H\;(\theta ,p)) \end{array}$$ (11)

thereby interpreting the log-posterior as the Hamiltonian

$$\begin{array}{c} H\;(\theta ,p)=-\text{log}\;(P\;(\theta ,p|\text{data})) \end{array}$$ (12)

where

$$\begin{array}{ccc} H\;(\theta ,p) & = & L\;(\theta )+V\;(\theta )+T\;(p). \end{array}$$ (13)

Here **p** = (*p*_1_, *p*_2_, …, *p*_*r*_) are the momenta, each corresponding to a parameter of interest, up to *r* parameters. In order to initialize the dynamics, we choose the value of these momenta at random from a standard normal distribution at the start of each HMC iteration. The pieces of the Hamiltonian function include the negative logarithm of the prior, *L*(*θ*) = −log (*P*(*θ*)), the negative logarithm of the likelihood, V(θ)=-log(P(D|θ)), and the kinetic energy given by,

$$\begin{array}{ccc} T(p) & = & \frac{pW^{-1}p}{2}, \end{array}$$ (14)

where *W* is a square mass matrix of size *r* × *r*.

To integrate the resulting Hamiltonian dynamics for analytical likelihoods we may use Stormer-Verlet (*i.e.*, plain leapfrog) [58, 59] which is the symplectic integrator of choice. However, to treat our non-analytic likelihoods, we resort to operator splitting, Strang-splitting [60].

In Strang-splitting, the Hamiltonian function is split into two components

$$\begin{array}{ccc} H(\theta ,p) & = & H^{1}(\theta ,p)+H^{2}(\theta ,p), \end{array}$$ (15)

where

$$\begin{array}{ccc} H^{1}(\theta ,p) & = & V(\theta ), \end{array}$$ (16)

and

$$\begin{array}{ccc} H^{2}(\theta ,p) & = & L(\theta )+T(p). \end{array}$$ (17)

With these two components, we apply the following three steps for each proposal within the HMC algorithm: 1) we simulate dynamics (integrate the Hamilton-Jacobi equations) within the parameter space for half a time-step according to *H*^1^(*θ*, **p**); 2) we advance a whole time-step according to *H*^2^(*θ*, **p**); and 3) we repeat the first step. By repeating these steps for a predetermined amount of time, we obtain a set of proposed parameter values, *θ*^*prop*^, and corresponding auxiliary variables, **p**^*prop*^, accepted or rejected according to a Metropolis-Hastings acceptance ratio, when compared to the set of parameters from the first step of the HMC, *θ*^*old*^ and variables **p**^*old*^. The acceptance probability reads

$$\begin{array}{ccc} R_{\theta^{old}}(\theta^{prop}) & = & \text{min}(1,\frac{e^{-H(\theta^{prop},p^{prop})}}{e^{-H(\theta^{old},p^{old})}}). \end{array}$$ (18)

Employing a combination of all three sampling methods discussed above, AMH, HMC, and PT, within our algorithm allows us to maximize the effectiveness of our sampling, enabling model inference on high dimensional, complicated biological systems from snapshot data without imposing *a priori* assumptions on the underlying system’s dynamics.

## 3 Results

Here for one and two state networks we benchmark alternative versions of our method: 1) the “full method”–our method using advanced proposal schemes (AMH and HMC) within the PT framework; 2) the “fixed proposals”–a version which generates proposals from a fixed proposal distribution (with no AMH and HMC) within the PT framework; 3) the “without PT”–a version containing advanced proposal schemes (AMH and HMC), sampling only a single Markov chain.

As we will show, even the full method may be inefficient for three state networks and beyond. Thus for the three state network, we contrast: 1) the hybrid “full method”, as before; and 2) the “full method + *γ*”–the full method, assisted by hand-specification of the product degradation rate, *γ*.

We demonstrate the sampling efficiency of each method by considering alternative methods along three dimensions: 1) accuracy of learned posteriors (that is, how close the learned posterior’s mode is to the ground truth) following a fixed number of samples; 2) duration of burn-in (the number of iterations to convergence); and 3) number of independent samples drawn as determined by the autocorrelation between successive samples. Here, we highlight selected results for the one, then two, and finally three state network from the analysis of synthetic snapshot data (for concreteness of the example data, chosen to replicate smFISH), and full results (plots containing information on all learned rates) are contained in section 1.3 in S1 Text.

We will find across all examples that the full method outperforms each of the opposing methods. Additionally, we observe that the stiffness of the CME renders a fixed proposal scheme infeasible even for the simplest case. Our advanced proposal schemes overcome the challenge encountered with fixed proposals by more frequently proposing parameters maintaining the stability of the eventual CME’s solution. Though it doesn’t prove vital in all cases, we repeatedly find that, since PT improves our method’s mixing by reducing the separation between the isolated modes of our posterior [53], it leads to faster convergence.

In all of our computational experiments, we will also find that AMH and HMC improve sampling efficiency for the reasons discussed in methods.

### 3.1 One state network

**Fig 2:**
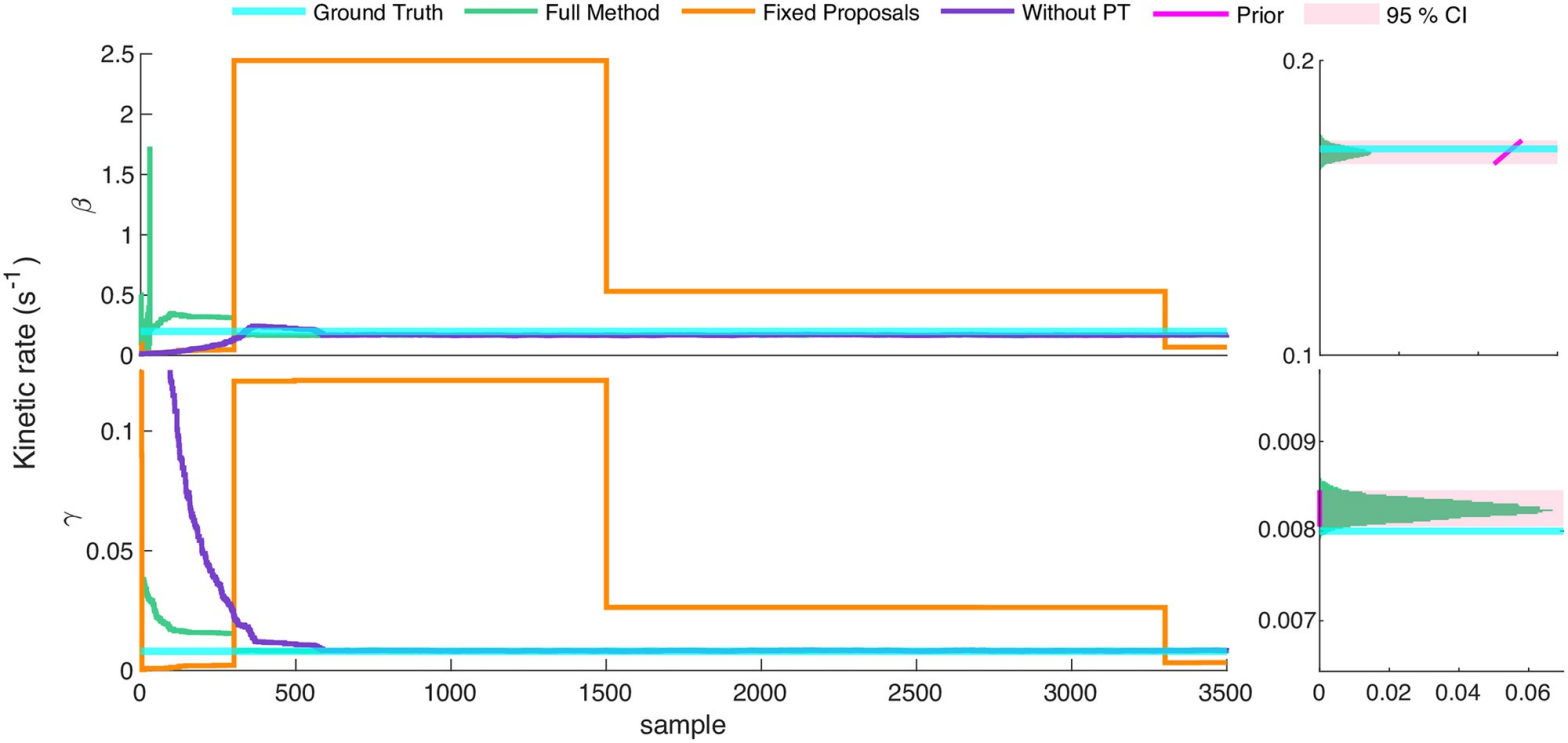
Inference strategies for the one state network. Here we show traces of MCMC samples drawn from the target distribution depicting the convergence of three methods outlined above. Clearly, the fixed proposal scheme (orange trace) demonstrates poor mixing for both parameters (*β*, production, and *γ*, degradation, rates). That is, it fails to propose samples which approach ground truth with as many or fewer samples as other methods. The performance difference between the full method (green trace) and the method without parallel tempering (purple trace) is less apparent, but still present for this example. To the right of each rate’s MCMC trace, we plot a histogram of the posterior for the corresponding parameter, complete with the ground truth (cyan line), prior density (magenta curve) and 95% confidence interval (magenta shaded region).

For ease of interpretation alone, here we show results of the parametric method (*i.e.*, fixing, as opposed to learning, the number of states). Fig 2 depicts traces of rates across MCMC samples converging in this case.

Fig 2 clearly shows the necessity of informed proposals for parameter inference on the one state network. Initializing from an uninformative prior, the full method converges to the ground truth first (within 500 iterations), while the method neglecting parallel tempering requires approximately 200 more iterations for convergence. Meanwhile, the method relying on a fixed distribution to generate proposals does not visit the ground truth rates within the number of samples allotted. Therefore though adaptive proposals are necessary for the one state network, PT is not entirely necessary to reach convergence in a reasonable number of samples, even if it does improve the speed of convergence.

**Fig 3:**
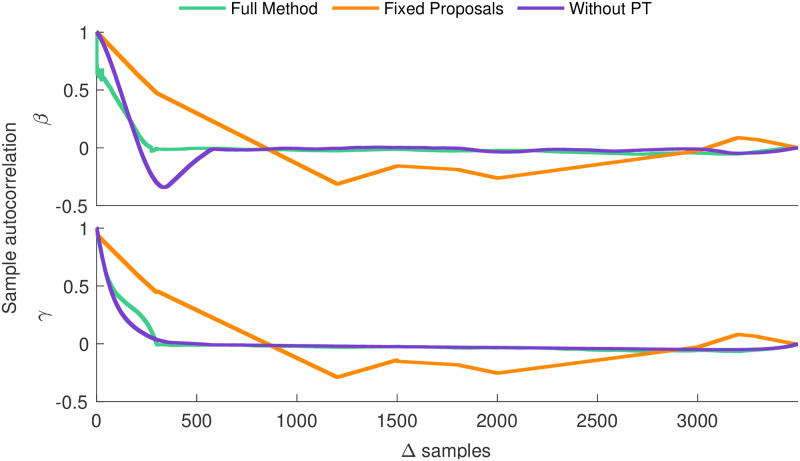
Sample autocorrelation analysis for the one state network. An auto-correlation that converges to zero fastest is ideal as it maximizes the number of independent samples. We see that the full method converges quickest.

The number of independent samples in an MCMC chain determines the fidelity of a Bayesian MCMC algorithm’s empirical characterization of its target distribution. Therefore lower autocorrelation (more **independence**) between samples allows characterization of the posterior in fewer MCMC samples. As indicated by Fig 3, the full method demonstrates a near immediate reduction in the sample autocorrelation for all parameters. Meanwhile, the method with uninformed proposals frequently rejects proposed models, and as a result of the negligible movement of the MCMC chain, fails to clearly indicate that successive samples are independent even over the entire duration of inference. Thus, the advanced proposal mechanisms improve efficiency by increasing the proportion of accepted samples.

**Fig 4:**
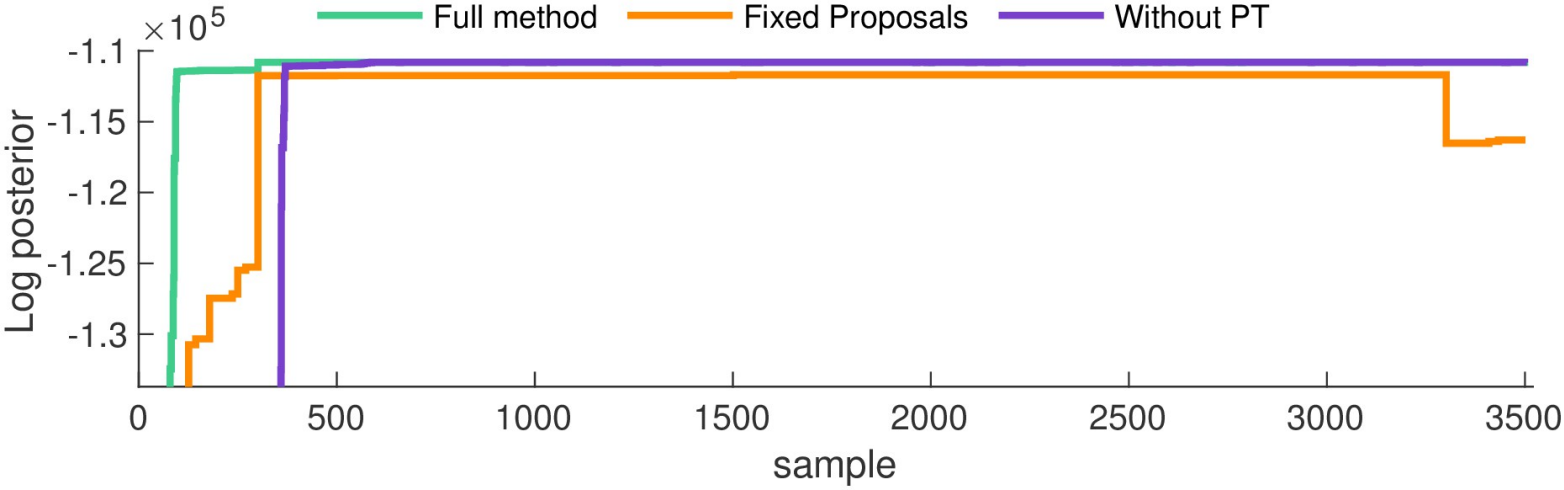
Convergence in the log-posterior for the one state network. Here we compare convergence of the log-posterior for the three methods outlined above. Echoing Fig 2, the full method demonstrates the fastest convergence, followed by the method without PT, with fixed proposals lagging behind.

Fig 4 shows that as each method approaches the ground truth network, its log-posterior increases. Predictably since it finds the ground truth network rates fastest, the full method’s log-posterior reaches its maximum first, and remains on its eventual ‘plateau’ for the highest number of MCMC iterations. In exact correspondence to Figs 2 and 3, the next fastest to converge is the method without PT, with the method with fixed proposals following.

Notably, a large decrease in the log-posterior occurs for the method with fixed proposals. This behavior is the result of repeatedly proposing improbable networks, a direct consequence of an MCMC method exploring the target distribution slowly.

### 3.2 Two state network

The remainder of analysis is concerned with the nonparametric inference method, learning the number of network nodes as well as rates.

**Fig 5:**
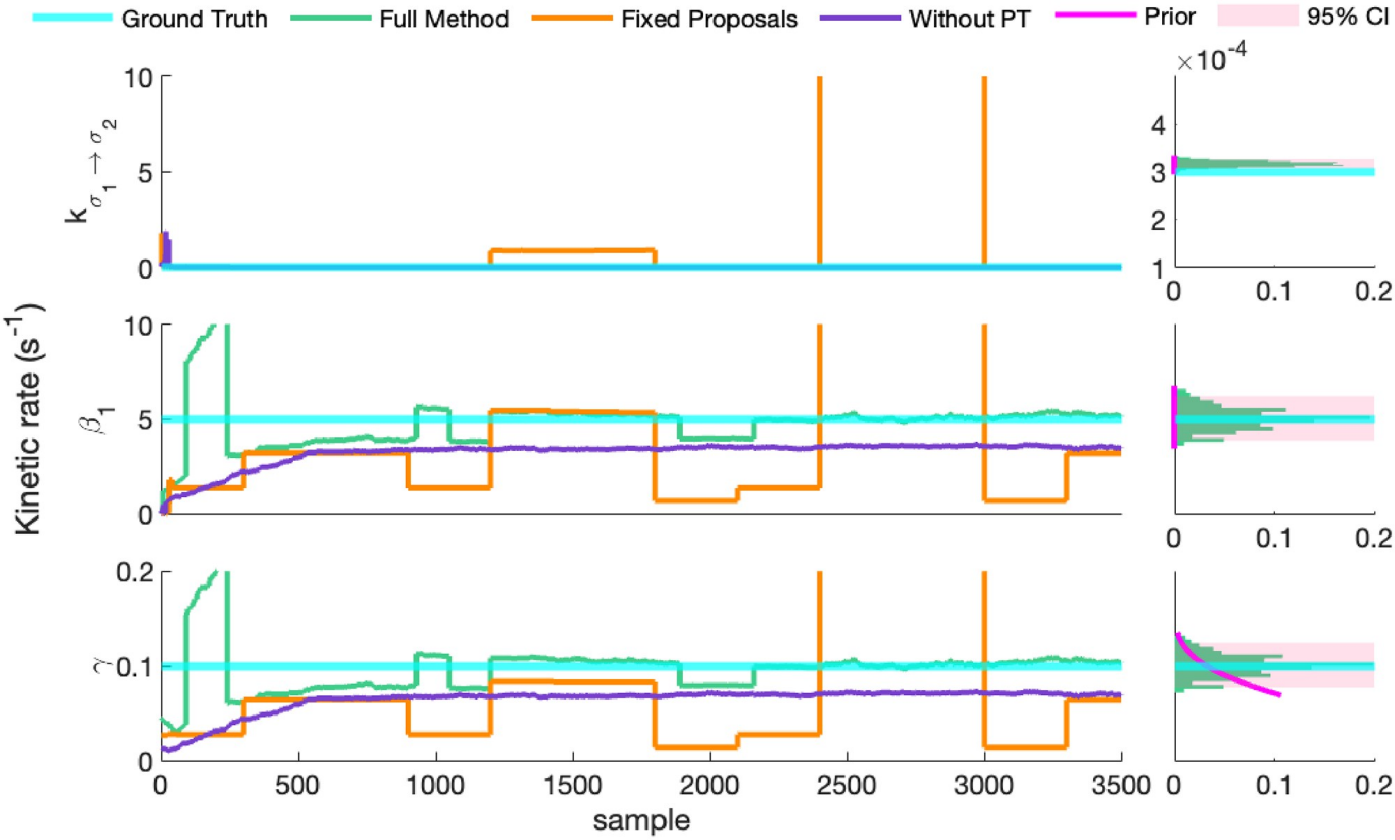
Inference strategies for the two state network. Here we compare our full method (green trace) to our method with PT removed (purple trace), and our method with adaptive proposals removed (orange trace). We show only representative rates though the rest are shown alongside our posterior over the number of states in Fig A in S1 Text. To demonstrate that the method reliably converges to the ground truth, the second column depicts marginal rate histograms (green bars) taken from the displayed MCMC chain as well as 5 additional MCMC chains, initialized from the prior. Additionally, to illustrate the method’s confidence, we depict (magenta shaded area) the region in which 95% of MCMC samples fall, and to demonstrate the distinctness of the posterior from the prior, we depict the rate prior density (magenta curve) within the region covered by the learned posterior.

Once again, Fig 5 demonstrates the necessity of the full method to accurately infer the two state network model in a reasonable number of samples. By the end of Fig 5’s 3500 samples, the full method has converged to the ground truth and is exploring the neighborhood of the ground truth, while the alternative methods lag behind. As we have seen earlier in our discussion of section 3.1, owing to its ability to swap chains (thereby effectively “jumping” around the space of models, potentially between isolated modes of the target distribution), the method which uses PT without advanced proposal mechanisms explores a broader model diversity than that without PT. In fact, the utility of the PT scheme is on full display in Fig 5, as the method without PT demonstrates trapping in a local posterior maximum that, even with AMH and HMC, requires PT to escape.

**Fig 6:**
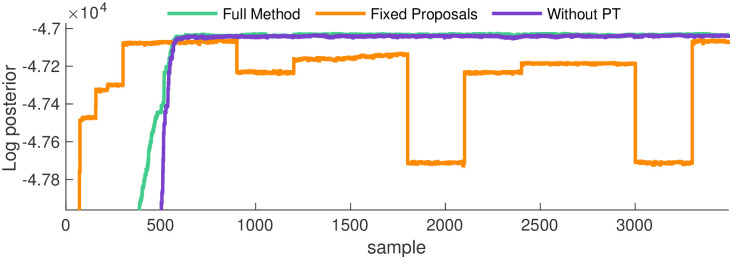
Convergence in the log-posterior for the two state network. Here we compare increases in the log-posterior for the three methods discussed so far. Echoing Fig 2, the full method demonstrates the fastest convergence, followed by the method without PT, with fixed proposals lagging behind. Examining the purple trace, the method without PT apparently converges but, due to local maximization, never reaches the ground truth parameters. We show autocorrelations for only the rates highlighted here, but the rest are shown in Fig B in S1 Text.

Analysis of the log-posterior clearly shows the local trapping of the method without PT on one of the target’s modes. Its log-posterior, the purple trace in Fig 6, flattens around iteration number 600 (notably, almost the exact same iteration as the full method), but it remains at values bested only slightly by the full method for the same reasons identified in section 3.1. This indicates that even though the method without PT “converges”, the quality of convergence is inferior to the full method, as shown in Fig 5.

### 3.3 Three state network

**Fig 7:**
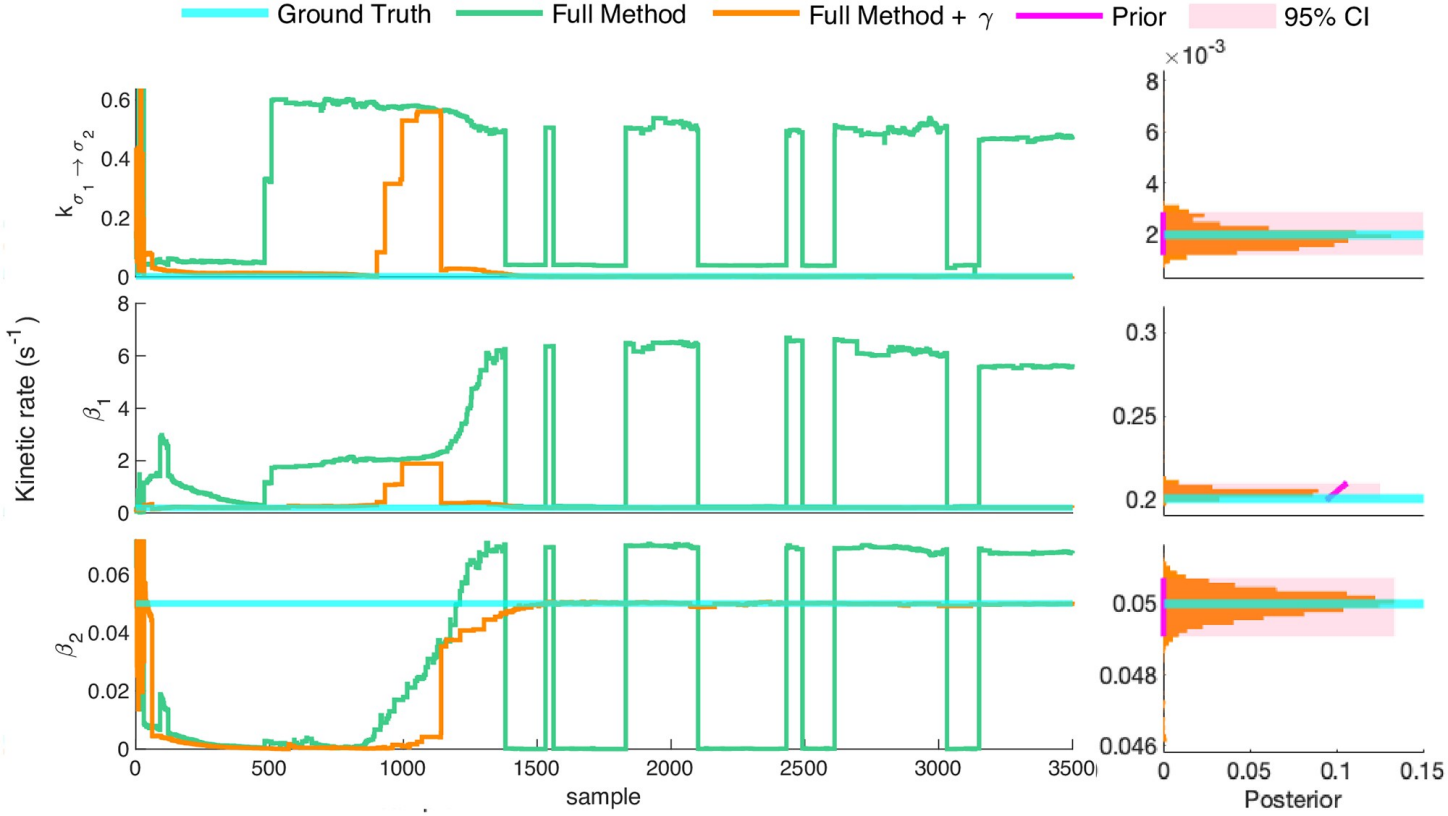
Nonparametric inference strategies for the three state network. Here we show traces depicting the convergence of two methods outlined above. Clearly, the full method (green trace) is insufficient, transitioning between local maxima rather than converging to the ground truth. By contrast, addition of a pre-calibrated degradation rate permits accurate inference in a timely manner. As before, we depict the 95% confidence interval (magenta shaded), and we depict the rate prior density (magenta curve) within the region covered by the learned posterior. What is more, we show results for all remaining rates alongside our posterior over the number of states in Fig C in S1 Text.

For the three state network, the dimensionality of the model space causes two problems: 1) the number of plausible candidate models is high, increasing the possibility for local trapping of the sampler; 2) owing to its high dimensionality, the model space is inherently difficult to explore. The influence of these issues is clear in Fig 7, where the full method fails to reach the ground truth rates after 3500 samples, since the snapshot data’s scant dynamical information results in a tendency toward trapping in local posterior maxima. Given enough time, the data is often sufficient to determine the relative strength of multiple local maxima using the full method and recover the ground truth for all parameters simultaneously (see supplemental figure 1 of [8]). We improve computational efficiency, we may reduce model dimensionality by fixing one or more parameters. Owing to its “global” role in the reaction network the degradation rate (*γ*) is a natural choice. In part, this is because production rate (*β*) can be reduced to compensate for a lower assumed degradation rate giving rise to approximate model indeterminacy.

Figs 7 and E in S1 Text demonstrate how specifying *γ* results in timely convergence of our nonparametric model inference method. This is further supported by Fig D in S1 Text which shows reduced autocorrelations, and thus more efficient sampling, under specification of *γ*.

## 4 Discussion

We have systematically characterized the performance of various different methods to learn the number of nodes and connectivities of reaction networks. For the purpose of analysis, we’ve used synthetic data inspired by smFISH, an essential tool in the study of gene transcription dynamics [61, 62]. Previously, attempts to quantify transcription dynamics have been hindered by two issues: 1) prohibitively costly likelihood calculations, and high dimensional parameter spaces; as well as 2) highly correlated and scale-separated parameters. Filling this gap, our method can learn models quickly and precisely. Owing to the issues we’ve identified, previous methods have resorted to assuming *a priori* that certain reaction pathways do not occur (*e.g.*, assuming that gene states are linearly or cyclically connected) [26, 36].

We improve upon the limitations of previous rate specification tools [3, 25, 25, 28, 34, 63] by considering advantages and disadvantages of combinations of HMC, PT, and AMH to a custom Gibbs sampling scheme. We utilize the resulting increased efficiency to sample complex posteriors inherent when simultaneously sampling networks and parameters within a Bayesian nonparametric paradigm.

As it stands, our method represents a distinct advancement in MCMC to quantify reaction networks. Notably, this advancement comes from combining previously-developed samplers albeit on a novel nonparametric framework requiring simultaneous inference of discrete and continuous parameters, rather than necessarily providing a distinct innovation in any one sampling technique. While we have explored three samplers, a number of other samplers exist (such as affine invariant ensemble samplers [64, 65], and pre-conditioned Monte Carlo [66]), though these may break down for high-dimensional [67] or non-convex [68] posteriors, respectively.

When HMC, AMH and PT are used in concert, these tools consistently provide a significant reduction in the number of iterations to reach convergence, compared to the same method with any of the three tools removed. Additionally analysis of sample auto-correlation (Fig 3, and Figs B and D in S1 Text) demonstrates our full method’s lower sample auto-correlation as compared to its alternatives. This result indicates that, in addition to converging in fewer samples, the number of independent samples per MCMC sample is greatest for the full method.

By further investigating limitations of advanced samplers, our tools allow us to set limits on the complexity of reaction networks that we can realistically learn from typical finite snapshot data set sizes before encountering severe albeit approximate model indeterminacy. For example in the three state gene network case, simply introducing additional data on RNA degradation into a joint likelihood with our RNA snapshot data partially resolves indeterminacy. Critically however, our method allows us to increase the amount of data considered to discover more complex networks while avoiding overfitting.

Although we developed our method with non-equilibrium data in mind, we can readily model measurements at equilibrium whose likelihood follows from a reaction network’s steady-state CME solution. In this case, the finite state projection (FSP) we use to compute likelihoods [69] may require, as an approximation, the introduction of an absorbing state [70]. Barring this modification to the generator matrix, sampling methods compared here treat equilibrium and non-equilibrium systems in precisely the same manner and their performance is not affected by the interpretation of the states at hand.

To our already general method, some additional generalizations are possible at little to no additional cost. For instance, we make the simplifying assumption here that data arrives uncorrupted by measurement noise. Adding any arbitrary emission distribution to our model merely entails the addition of a distribution over different underlying RNA counts and marginalization over these counts [8]. Along these same lines, the initial condition used to solve the CME can be modified to suit any experimentally relevant initial condition.

Moving forward, provided appropriate data, our method lends itself to simple adaptation to infer alternative reaction networks, not necessarily composed of gene states with associated RNA production, by simply modifying the structure of the generator matrix (see section 1.2 in S1 Text).

To help further improve computational efficiency, we may use genetic algorithms [25] to initiate our MCMC chain closer to the global posterior maximum than what is currently done by initializing samples from the prior. In this manner, burn-in time might be reduced, though local-maximum trapping remains unavoidable. We exclude this technique here for two reasons: 1) the genetic algorithm employed in [25] requires pre-specification of the number of gene states, a limitation that our method overcomes; 2) analyzing the characteristics and duration of burn-in are of primary interest here.

While introducing additional cost, we can extend our framework to consider more complex reaction networks. First, we may relax our assumption that only a single transcribing copy of each gene exists in every cell, and instead we may learn a distribution over the number of genes in each cell in the case genes appear on plasmids. Additionally, time-varying rates of production, state transitions, or degradation, [3, 26, 71] are readily added to the method, at the modest cost of introducing time-dependency to the generator matrix. The generator matrix may be further expanded to consider RNA transport across spatial compartments such as the nucleus or cytoplasm [3]. Finally, as the number of quantified genes increases using multiplexed smFISH methods [72], with some modification, our approach allows for models of co-varying gene expression networks [62, 72, 73].

Each generalization mentioned above introduces complexity to the likelihood’s computation arising from the increase in the state number and complexity of the connectivity map. Obtaining computationally efficient CME solutions in deriving the likelihood, the key computational bottleneck, is therefore critical in implementing these generalizations. Currently, the time cost of the likelihood computation scales roughly linear with **A**’s dimension, with evidence that alternative methods to integrate the CME—*i.e.*, FSP based Krylov subspace methods [74, 75]—may be faster than the CME solution method used here. How the computational cost of computing **A**’s CME solution scales with its sparsity is more complex. In the case of densely connected reaction networks, the recently proposed Quantized Tensor Train method [76] may be more efficient than the FSP-based Krylov subspace approach which uses incremental time stepping rather than jumping immediately to the times desired for analysis. Alternatively, there have been promising attempts to solve ODEs using neural networks [77] or to parallelize matrix exponentiation using GPU hardware [78]. In addition to facilitating the difficulties arising from dense CME generator matrix exponentiation, neural network approaches may further enable parameter inference models when non-Markovian dynamics are present [79]. These tools may eventually become important as we move to monitoring dynamics in real time and deal with, for instance, finite time fluorescent protein maturation introducing delays between gene expression and fluorescence reporter detections [80].

## Supporting information

S1 Text Supporting information file containing Supporting Figures (Fig A—E) and Supporting Table (Table A). Fig A. Two state nonparametric network inference strategies. Fig B. Two state nonparametric network inference autocorrelations. Fig C. Three state nonparametric network inference strategies. Fig D. Sample autocorrelation analysis of three state network. Fig E. Convergence in the log-posterior for the three state network. Table A. Table of symbol names and (where applicable) their numerical values.
(PDF)
