# Supplementary material for: Monte Carlo samplers for efficient network inference
Source: PLoS Comput Biol. 2023 Jul 18;19(7):e1011256. doi: 10.1371/journal.pcbi.1011256 (PMC10353823; doi:10.1371/journal.pcbi.1011256)
Supplement: S1 Text — Supporting information file containing Supporting Figures (Fig A—E) and Supporting Table (Table A). Fig A. Two state nonparametric network inference strategies. Fig B. Two state nonparametric network inference autocorrelations. Fig C. Three state nonparametric network inference strategies. Fig D. Sample autocorrelation analysis of three state network. Fig E. Convergence in the log-posterior for the three state network. Table A. Table of symbol names and (where applicable) their numerical values. (PDF) [file pcbi.1011256.s001.pdf]

1 Supporting information

1.1 Model Interpretations

Here we expand upon the models described in section 4. The reaction network discussed in section 5 is motivated by two experimental pathways, which each produce snapshot data. Precisely the same biochemical reaction network can describe both experiments, and switching between the two is a simple matter of re-interpreting the physical meaning of nodes and rates. We describe these two interpretations below.

Gene Expression The simplest reaction network examined in section 5 is the one state network model of gene expression. In this model, RNA molecules transcribe stochastically at rate, β, from a single gene state, σ_1, and degrade stochastically at rate, γ. As with the following models, we refer to all kinetic parameters collectively as θ. Here, $\theta = (\beta, \gamma)$. Alternatively, RNA might transcribe at one of two production rates: β_1 or β_2, based on the state of the gene, σ_1 or σ_2. Therefore, the two state model is specified by, β_1, β_2, γ, and the rates at which the gene transitions between its states, $k_{\sigma_1 \to \sigma_2}$ and $k_{\sigma_2 \to \sigma_1}$. Thus for the two state model, $\theta = (\beta_1, \gamma, k_{\sigma_1 \to \sigma_2}, k_{\sigma_2 \to \sigma_1})$. Subsequent models of L gene states will then follow in kind, with β_1,$\cdots$,β_L,γ and transition rates $k_{\sigma_i \to \sigma_j}$ for $i \neq j$ and $i, j = 1, \ldots, L$ specifying the model completely.

Allosteric Control When evaluating the case of allosterically controlled enzyme activity, we must simply reinterpret the physical meaning of the rates. Here, transition rates $k_{\sigma_i \to \sigma_j}$ correspond to promoter/inhibitor binding, conformational transitions, or any other reaction which may change substrate binding affinity . Thereby, state transitions will change the rate at which product emerges, alternating between β_i. Then, as in the previous case the degradation rate γ represents the rate at which the product either degrades or becomes inaccessible to the imaging method in use.

1.2 The Likelihood

1.2.1 One State Network

In the simplest case, the one state model, the state space of the system ranges over all possible product counts, $\mathcal{M} = \{0, 1, \ldots, M\}$, with M the necessary assumed maximum number of product copies, is chosen larger than the maximum number observed. For this model, the generator matrix, here $\mathbb{A}_1$, is a square matrix whose size is dictated by the maximum single cell product count, denoted by M. $\mathbb{A}_1$ has the structure:

$$\mathbb{A}_1 = \begin{pmatrix} -\beta_1 & \gamma & 0 & \cdots & 0 \\ \beta_1 & -(\beta_1+\gamma) & 2\gamma & \ddots & \vdots \\ 0 & \beta_1 & -(\beta_1+2\gamma) & \ddots & 0 \\ \vdots & \ddots & \ddots & \ddots & M\gamma \\ 0 & \cdots & 0 & \beta_1 & -(\beta_1+M\gamma) \end{pmatrix}. \tag{19}$$

With this generator matrix, we can compute the solution, $P_\theta^t(m)$, of the CME Eq. 2, and

$$\mathbf{P}(t|\theta) = \left(P_\theta^t(0), P_\theta^t(1), \ldots, P_\theta^t(M)\right)^T. \tag{20}$$

The solution to the CME feeds into our likelihood, $P(\bar{\bar{m}}|\theta)$, by taking into account the fact that the single cell RNA counts are independent and identically distributed.

Thus the likelihood for the one state model is

$$P\left(\bar{\bar{m}}|\theta\right) = \prod_{k=1}^{K}\prod_{j=1}^{J_k} P_\theta^{t_k}\left(m_k^j\right). \tag{21}$$

1.2.2 Multi-state gene network

In the multi-state case, the gene may occupy multiple states represented by $\mathcal{S} = \{\sigma_1, \cdots, \sigma_L\}$, where L is the number of gene states. The space of product counts remains $\mathcal{M} = \{0, 1, \ldots, M\}$. Therefore, the system's state space becomes $\mathcal{S} \times \mathcal{M}$ and the CME solution becomes $P_\theta^t(\sigma_i, m)$, representing the probability of the gene state being in state σ_i, for $i = 1, 2, \cdots, L$, with m product copies at time t, given parameters θ.

The full generator matrix for the L state model, in block form becomes:

$$\mathbf{A}^{(L)} = \begin{pmatrix} \mathbb{A}_1 - \sum\limits_{\ell \neq 1} \mathbf{K}_{1\ell} & \mathbf{K}_{21} & \cdots & \mathbf{K}_{L1} \\ \mathbf{K}_{12} & \mathbb{A}_2 - \sum\limits_{\ell \neq 2} \mathbf{K}_{2\ell} & \cdots & \cdots \\ \vdots & \ddots & \ddots & \mathbf{K}_{LL-1} \\ \mathbf{K}_{1L} & \cdots & \mathbf{K}_{L-1L} & \mathbb{A}_L - \sum\limits_{\ell \neq L} \mathbf{K}_{L\ell} \end{pmatrix},$$

with $\mathbb{A}_\ell$, as defined in 19, now indexed to identify the replacement everywhere of β_1 with β_ℓ, and

$$\mathbf{K}_{i\ell} \equiv k_{\sigma_i \to \sigma_\ell} \cdot \mathbb{I}_{M+1}. \tag{22}$$

The CME is given again by Eq. 2, however, now

$$\mathbf{P}\left(t|\theta\right) = \left(P_\theta^t\left(\sigma_1, 0\right), P_\theta^t\left(\sigma_1, 1\right), \ldots, P_\theta^t\left(\sigma_1, M\right), P_\theta^t\left(\sigma_2, 0\right), P_\theta^t\left(\sigma_2, 1\right), P_\theta^t\left(\sigma_2, M\right)\right)^T, \tag{23}$$

and to construct our likelihood, we must marginalize over the state space for the gene:

$$P\left(\bar{\bar{m}}|\theta\right) = \prod_{k=1}^{K}\prod_{j=1}^{J_k}\left(\sum_{s_k^j = \sigma_1, \sigma_2} P_\theta^{t_k}\left(s_k^j, m_k^j\right)\right). \tag{24}$$

1.3 Supporting Figures

Here we show results for the rates omitted from the main text.

Two state

Three state

1.4 Table of Hyper-parameters

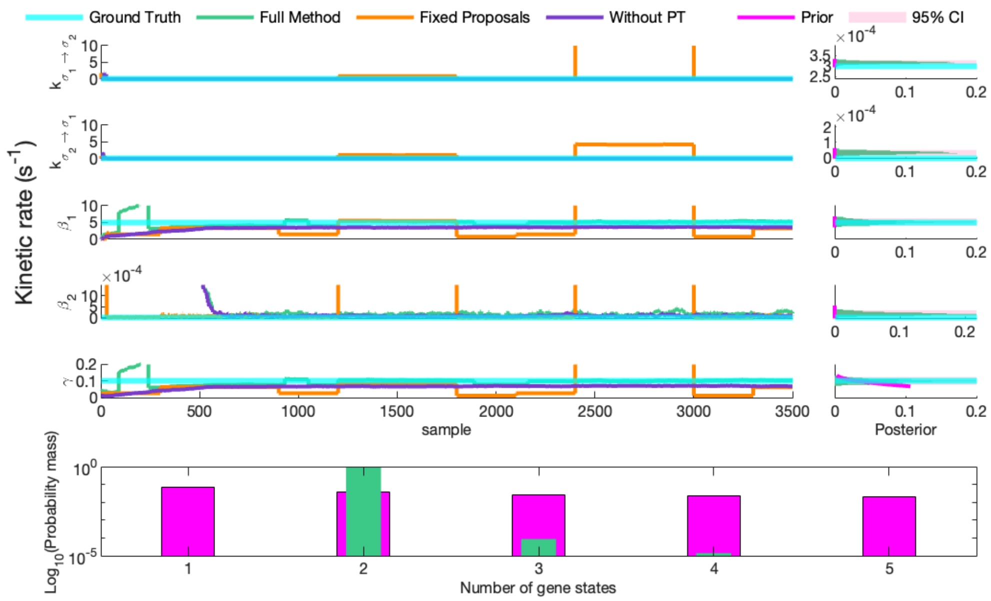

Fig A. Two state nonparametric network inference strategies. Corresponding to Fig 5, here, we show results for all rates in the two state network both as MCMC traces and marginal rate histograms (complete with 95% confidence regions). The bottom panel depicts our learned posterior over the number of gene states, superposed over the prior.

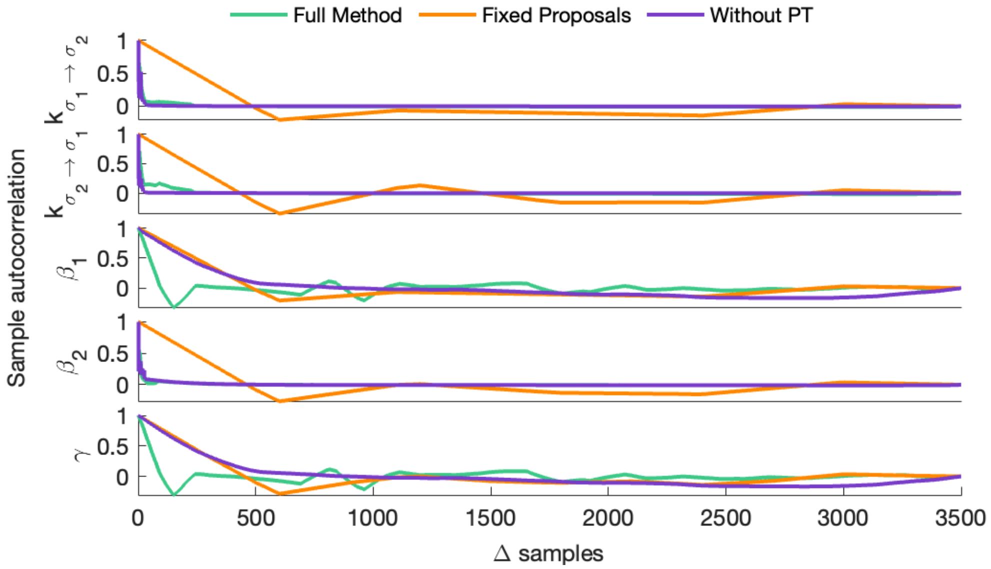

Fig B. Two state nonparametric network inference autocorrelations. Here, we show sample autocorrelations for all rates in the two state gene network.

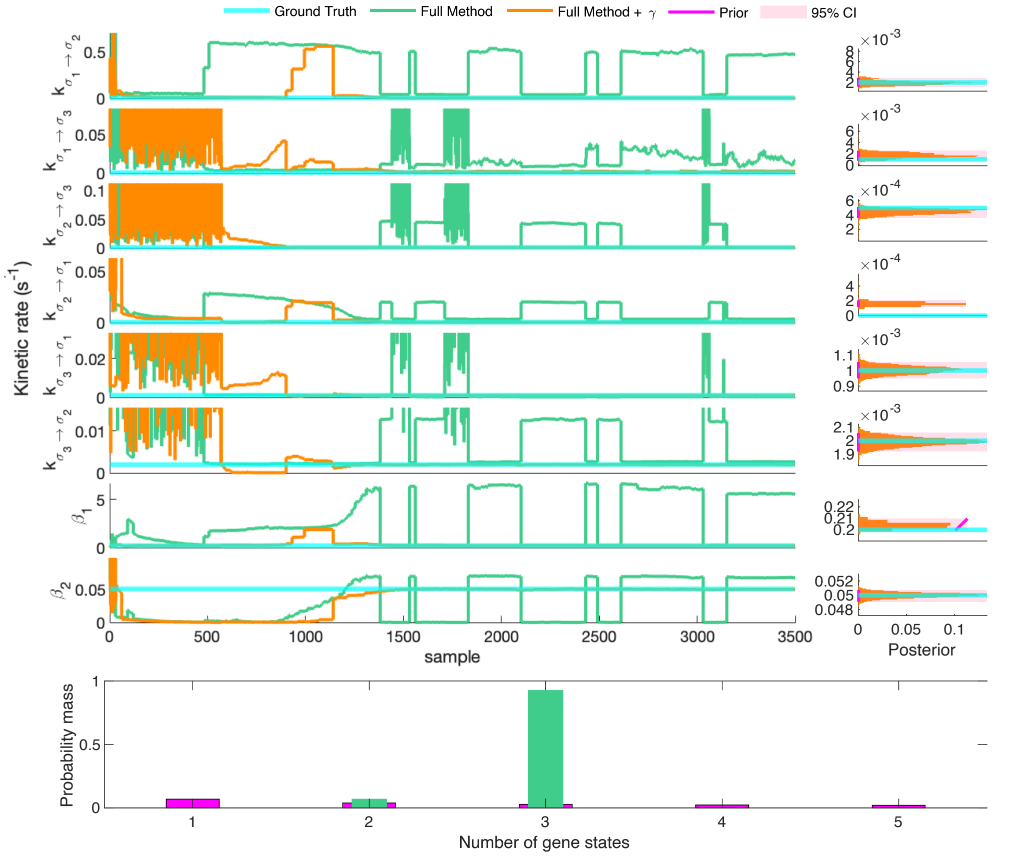

Fig C. Three state nonparametric network inference strategies. Corresponding to Fig 7, here, we show results for all rates in the three state network alongside our learned posterior over the number of gene states.

Parameter	Value	Description
t_f	3600 s	The duration of the synthetic RNA FISH experiment
J_k	2000 for all k	The number of RNA counts provided per time point
K	10	The number of time points (equally spaced over $0 - t_f$)
L	6	The weak limit on the number of gene states
ζ	5	The success probability prior hyper-parameter
ϵ	3.7508×10^{-17}	The small value multiplying the identity matrix for AMH
μ_p	0	The HMC momentum distribution mean
σ_p	1	The HMC momentum distribution standard deviation
α_M	2	The HMC mass distribution scale parameter
β_M	2	The HMC mass distribution shape parameter
μ_{rates}	-1	The prior mean on the log of rates
σ_{rates}	1	The prior standard deviation for rates

Table A

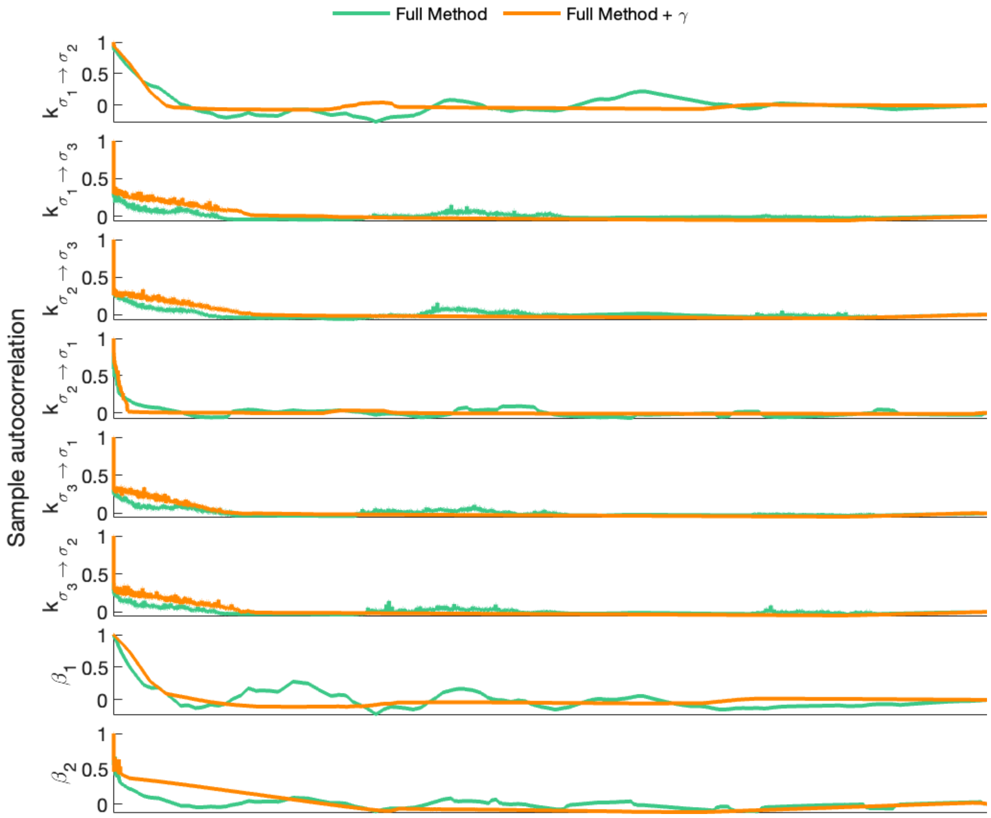

Fig D. Sample autocorrelation analysis of three state network. Traces of sample autocorrelation vs sample separation (Δ *samples*) for the three state network. Predictably, since they use the same sampling method, autocorrelations are comparable between the methods compared here.

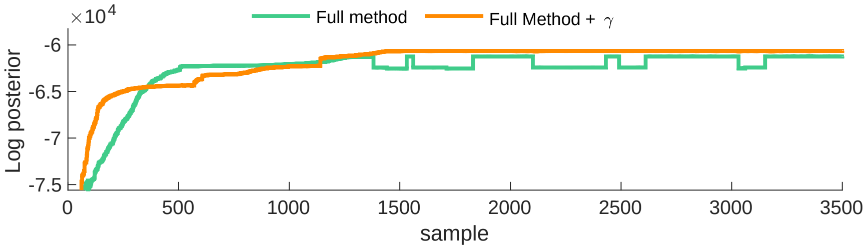

Fig E. Convergence in the log-posterior for the three state network. Here, we compare increases in the log-posterior for the two methods discussed so far. In correspondence with Fig 7, the log-posterior with specified degradation rate quickly surpasses the full method, as the full method becomes trapped between local maxima.

References

1. Hung KYS, Klumpe S, Eisele MR, Elsasser S, Tian G, Sun S, et al. Allosteric control of Ubp6 and the proteasome via a bidirectional switch. Nature communications. 2022;13(1):1–13.

2. Fletcher A, Zhao R, Enciso G. Non-cooperative mechanism for bounded and ultrasensitive chromatin remodeling. Journal of Theoretical Biology. 2022;534:110946.

3. Munsky B, Li G, Fox ZR, Shepherd DP, Neuert G. Distribution shapes govern the discovery of predictive models for gene regulation. Proceedings of the National Academy of Sciences. 2018;115:7533–7538.

4. Shen X, Wang R, Xiong X, Yin Y, Cai Y, Ma Z, et al. Metabolic reaction network-based recursive metabolite annotation for untargeted metabolomics. Nature communications. 2019;10(1):1–14.

5. Gatto F, Ferreira R, Nielsen J. Pan-cancer analysis of the metabolic reaction network. Metabolic engineering. 2020;57:51–62.

6. Liu B, Mavrova SN, van den Berg J, Kristensen SK, Mantovanelli L, Veenhoff LM, et al. Influence of fluorescent protein maturation on FRET measurements in living cells. ACS sensors. 2018;3(9):1735–1742.

7. Morisaki T, Lyon K, DeLuca KF, DeLuca JG, English BP, Zhang Z, et al. Real-time quantification of single RNA translation dynamics in living cells. Science. 2016;352(6292):1425–1429.

8. Kilic Z, Schweiger M, Moyer C, Shepherd D, Pressé S. Gene expression model inference from snapshot RNA data using Bayesian non-parametrics. Nature Computational Science. 2023; p. 1–10.

9. Fritsche-Guenther R, Witzel F, Sieber A, Herr R, Schmidt N, Braun S, et al. Strong negative feedback from Erk to Raf confers robustness to MAPK signalling. Molecular systems biology. 2011;7(1):489.

10. Femino AM, Fay FS, Fogarty K, Singer RH. Visualization of single RNA transcripts in situ. Science. 1998;280(5363):585–590.

11. Marzi MJ, Ghini F, Cerruti B, De Pretis S, Bonetti P, Giacomelli C, et al. Degradation dynamics of microRNAs revealed by a novel pulse-chase approach. Genome research. 2016;26(4):554–565.

12. Tak T, Wijten P, Heeres M, Pickkers P, Scholten A, Heck AJ, et al. Human CD62Ldim neutrophils identified as a separate subset by proteome profiling and in vivo pulse-chase labeling. Blood, The Journal of the American Society of Hematology. 2017;129(26):3476–3485.

13. Wang Z, Gerstein M, Snyder M. RNA-Seq: a revolutionary tool for transcriptomics. Nature reviews genetics. 2009;10:57–63.

14. Love MI, Huber W, Anders S. Moderated estimation of fold change and dispersion for RNA-Seq data with DESeq2. Genome biology. 2014;15:550.

15. Grabherr MG, Haas BJ, Yassour M, Levin JZ, Thompson DA, Amit I, et al. Full-length transcriptome assembly from RNA-Seq data without a reference genome. Nature biotechnology. 2011;29:644–652.

16. Ziegenhain C, Vieth B, Parekh S, Hellmann I, Enard W. Quantitative single-cell transcriptomics. Briefings in functional genomics. 2018;17:220–232.

17. Gaidatzis D, Burger L, Florescu M, Stadler MB. Analysis of intronic and exonic reads in RNA-Seq data characterizes transcriptional and post-transcriptional regulation. Nature biotechnology. 2015;33:722–729.

18. Rahman S, Zenklusen D. Single-molecule resolution fluorescent in situ hybridization (smFISH) in the yeast S. cerevisiae. In: Imaging Gene Expression. Springer; 2013. p. 33–46.

19. Shaffer SM, Wu MT, Levesque MJ, Raj A. Turbo FISH: a method for rapid single molecule RNA FISH. PloS one. 2013;8(9):e75120.

20. Asano SM, Gao R, Wassie AT, Tillberg PW, Chen F, Boyden ES. Expansion microscopy: protocols for imaging proteins and RNA in cells and tissues. Current protocols in cell biology. 2018;80(1):e56.

21. Kramer A, Calderhead B, Radde N. Hamiltonian Monte Carlo methods for efficient parameter estimation in steady state dynamical systems. BMC Bioinformatics. 2014;15(1):253. doi:10.1186/1471-2105-15-253.

22. Hellander A, Lötstedt P. Hybrid method for the chemical master equation. Journal of Computational Physics. 2007;227(1):100–122.

23. Peleš S, Munsky B, Khammash M. Reduction and solution of the chemical master equation using time scale separation and finite state projection. The Journal of chemical physics. 2006;125(20):204104.

24. Neuert G, Munsky B, Tan RZ, Teytelman L, Khammash M, Van Oudenaarden A. Systematic identification of signal-activated stochastic gene regulation. Science. 2013;339:584–587.

25. Vo HD, Fox Z, Baetica A, Munsky B. Bayesian estimation for stochastic gene expression using multifidelity models. The Journal of Physical Chemistry B. 2019;123:2217–2234.

26. Wang M, Zhang J, Xu H, Golding I. Measuring transcription at a single gene copy reveals hidden drivers of bacterial individuality. Nature microbiology. 2019;4:2118–2127.

27. Munsky B, Fox Z, Neuert G. Integrating single-molecule experiments and discrete stochastic models to understand heterogeneous gene transcription dynamics. Methods. 2015;85:12–21.

28. Neuert G, Munsky B, Tan RZ, Teytelman L, Khammash M, van Oudenaarden A. Systematic Identification of Signal-Activated Stochastic Gene Regulation. Science. 2013;339(6119):584–587. doi:10.1126/science.1231456.

29. Cheng Y, Li D, Jiang W. The Exact Inference of Beta Process and Beta Bernoulli Process From Finite Observations. Computer Modeling in Engineering & Sciences. 2019;121:49–82.

30. Thibaux R, Jordan MI. Hierarchical Beta processes and the Indian buffet process. In: Artificial Intelligence and Statistics; 2007. p. 564–571.

31. Sgouralis I, Bryan JS, Presse S. Enumerating High Numbers of Fluorophores from Photobleaching Experiments: a Bayesian Nonparametrics Approach. bioRxiv. 2020;.

32. Tavakoli M, Jazani S, Sgouralis I, Shafraz OM, Sivasankar S, Donaphon B, et al. Pitching single-focus confocal data analysis one photon at a time with Bayesian nonparametrics. Physical Review X. 2020;10(1):011021.

33. Jazani S, Sgouralis I, Shafraz OM, Levitus M, Sivasankar S, Pressé S. An alternative framework for fluorescence correlation spectroscopy. Nature communications. 2019;10(1):1–10.

34. Lin YT, Buchler NE. Exact and efficient hybrid Monte Carlo algorithm for accelerated Bayesian inference of gene expression models from snapshots of single-cell transcripts. The Journal of chemical physics. 2019;151:024106.

35. Wolff U, Collaboration A, et al. Monte Carlo errors with less errors. Computer Physics Communications. 2004;156(2):143–153.

36. Li G, Neuert G. Multiplex RNA single molecule FISH of inducible mRNAs in single yeast cells. Scientific data. 2019;6:1–9.

37. Modi T, Ozkan SB, Pressé S. Information Propagation in Time through Allosteric Signaling. Biophysical Journal. 2021;120(3):300a.

38. Schuh L, Saint-Antoine M, Sanford EM, Emert BL, Singh A, Marr C, et al. Gene Networks with Transcriptional Bursting Recapitulate Rare Transient Coordinated High Expression States in Cancer. Cell Systems. 2020;10:363 – 378.e12.

39. Golding I, Paulsson J, Zawilski SM, Cox EC. Real-time kinetics of gene activity in individual bacteria. Cell. 2005;123:1025–1036.

40. So Lh, Ghosh A, Zong C, Sepúlveda LA, Segev R, Golding I. General properties of transcriptional time series in Escherichia coli. Nature genetics. 2011;43:554–560.

41. Gelman A, Carlin JB, Stern HS, Rubin DB. Bayesian data analysis. Chapman and Hall/CRC; 1995.

42. Hastings WK. Monte Carlo sampling methods using Markov chains and their applications. Biometrika. 1970;57(1):97–109.

43. Smith AFM, Roberts GO. Bayesian Computation Via the Gibbs Sampler and Related Markov Chain Monte Carlo Methods. J Roy Stat Soc B. 1993;55(1):3–23.

44. Metropolis N, Rosenbluth AW, Rosenbluth MN, Teller AH, Teller E. Equation of state calculations by fast computing machines. The journal of chemical physics. 1953;21(6):1087–1092.

45. Sgouralis I, Pressé S. An introduction to infinite HMMs for single-molecule data analysis. Biophysical journal. 2017;112(10):2021–2029.

46. Sgouralis I, Pressé S. Icon: an adaptation of infinite hmms for time traces with drift. Biophysical journal. 2017;112:2117–2126.

47. Sgouralis I, Madaan S, Djutanta F, Kha R, Hariadi RF, Pressé S. A Bayesian nonparametric approach to single molecule forster resonance energy transfer. The Journal of Physical Chemistry B. 2018;123(3):675–688.

48. Berg BA. Introduction to Markov chain Monte Carlo simulations and their statistical analysis. Markov Chain Monte Carlo Lect Notes Ser Inst Math Sci Natl Univ Singap. 2005;7:1–52.

49. Gupta S, Lee RE, Faeder JR. Parallel Tempering with Lasso for model reduction in systems biology. PLoS computational biology. 2020;16(3):e1007669.

50. Earl DJ, Deem MW. Parallel tempering: Theory, applications, and new perspectives. Physical Chemistry Chemical Physics. 2005;7(23):3910–3916.

51. Fukunishi H, Watanabe O, Takada S. On the Hamiltonian replica exchange method for efficient sampling of biomolecular systems: Application to protein structure prediction. The Journal of chemical physics. 2002;116(20):9058–9067.

52. Gupta S, Hainsworth L, Hogg J, Lee R, Faeder J. Evaluation of parallel tempering to accelerate Bayesian parameter estimation in systems biology. In: 2018 26th Euromicro International Conference on Parallel, Distributed and Network-based Processing (PDP). IEEE; 2018. p. 690–697.

53. Neal RM. MCMC using Hamiltonian dynamics; 2012.

54. Neal RM, et al. MCMC using Hamiltonian dynamics. Handbook of markov chain monte carlo. 2011;2(11):2.

55. Haario H, Saksman E, Tamminen J, et al. An adaptive Metropolis algorithm. Bernoulli. 2001;7:223–242.

56. Andrieu C, Thoms J. A tutorial on adaptive MCMC. Statistics and computing. 2008;18(4):343–373.

57. Betancourt M. A Conceptual Introduction to Hamiltonian Monte Carlo; 2017.

58. Dimova S, Bazlyankov T. Numerical methods for Hamiltonian systems: Implementation and comparison. In: AIP Conference Proceedings. vol. 1684. AIP Publishing LLC; 2015. p. 090002.

59. Verlet L. Computer" experiments" on classical fluids. I. Thermodynamical properties of Lennard-Jones molecules. Physical review. 1967;159(1):98.

60. Strang G. On the construction and comparison of difference schemes. SIAM Journal on Numerical Analysis. 1968;5(3):506–517.

61. Raj A, Van Den Bogaard P, Rifkin SA, Van Oudenaarden A, Tyagi S. Imaging individual mRNA molecules using multiple singly labeled probes. Nature methods. 2008;5:877–879.

62. Wheat JC, Sella Y, Willcockson M, Skoultchi AI, Bergman A, Singer RH, et al. Single-molecule imaging of transcription dynamics in somatic stem cells. Nature. 2020;583(7816):431–436.

63. Ballnus B, Schaper S, Theis FJ, Hasenauer J. Bayesian parameter estimation for biochemical reaction networks using region-based adaptive parallel tempering. Bioinformatics. 2018;34:i494–i501.

64. Foreman-Mackey D, Hogg DW, Lang D, Goodman J. emcee: the MCMC hammer. Publications of the Astronomical Society of the Pacific. 2013;125(925):306.

65. Goodman J, Weare J. Ensemble samplers with affine invariance. Communications in applied mathematics and computational science. 2010;5(1):65–80.

66. Karamanis M, Beutler F, Peacock JA, Nabergoj D, Seljak U. Accelerating astronomical and cosmological inference with preconditioned Monte Carlo. Monthly Notices of the Royal Astronomical Society. 2022;516(2):1644–1653.

67. Huijser D, Goodman J, Brewer BJ. Properties of the affine-invariant ensemble sampler's 'stretch move'in high dimensions. Australian & New Zealand Journal of Statistics. 2022;64(1):1–26.

68. Efendiev Y, Hou T, Luo W. Preconditioning Markov chain Monte Carlo simulations using coarse-scale models. SIAM Journal on Scientific Computing. 2006;28(2):776–803.

69. Munsky B, Khammash M. The finite state projection algorithm for the solution of the chemical master equation. The Journal of chemical physics. 2006;124(4):044104.

70. Gupta A, Khammash M. Finding the steady-state solution of the chemical master equation. In: 2017 IEEE Conference on Control Technology and Applications (CCTA); 2017. p. 953–954.

71. Weber L, Raymond W, Munsky B. Identification of gene regulation models from single-cell data. Physical biology. 2018;15:055001.

72. Chen KH, Boettiger AN, Moffitt JR, Wang S, Zhuang X. Spatially resolved, highly multiplexed RNA profiling in single cells. Science. 2015;348(6233):aaa6090.

73. Lubeck E, Coskun AF, Zhiyentayev T, Ahmad M, Cai L. Single-cell in situ RNA profiling by sequential hybridization. Nature methods. 2014;11(4):360.

74. Vo H, Sidje RB. Improved Krylov-FSP method for solving the chemical master equation. Lect Notes Eng Comput Sci. 2016;2226.

75. Vo HD, Munsky BE. A parallel implementation of the Finite State Projection algorithm for the solution of the Chemical Master Equation. bioRxiv. 2020;.

76. Kazeev V, Khammash M, Nip M, Schwab C. Direct solution of the chemical master equation using quantized tensor trains. PLoS computational biology. 2014;10(3):e1003359.

77. Dufera TT. Deep neural network for system of ordinary differential equations: Vectorized algorithm and simulation. Machine Learning with Applications. 2021; p. 100058.

78. Defez E, Ibáñez J, Alonso-Jordá P, Alonso JM, Peinado J. On Bernoulli matrix polynomials and matrix exponential approximation. Journal of Computational and Applied Mathematics. 2022;404:113207.

79. Jiang Q, Fu X, Yan S, Li R, Du W, Cao Z, et al. Neural network aided approximation and parameter inference of non-Markovian models of gene expression. Nature communications. 2021;12(1):1–12.

80. Dong GQ, McMillen DR. Effects of protein maturation on the noise in gene expression. Physical Review E. 2008;77(2):021908.
